# Antipyrin in Acute Bronchitis of Children

**Published:** 1888-04

**Authors:** 


### *ANTIPYRIN IN ACUTE BRONCHITIS OF CHILDREN.*

For some months, innumerable articles have appeared in current medical literature discussing the physiological and therapeutic action of antipyrin. That the drug has a wide range of usefulness, is an undoubted fact. There is little doubt but that the drug will obtain a permanent place in therapeutics, but we need much more exact information in regard to the indications for its use. It is surprising, as Friedlander points out in a late number of the *Therapeutische Monatshefte,* that among so many articles describing the action of the drug, so few have appeared which consider its usefulness in the diseases of children. Friedlander has observed with care the indications by which the usefulness of the drug can be gauged in individual cases of acute bronchitis in children.

If the cases of bronchitis are classified according to their amenability to antipyrin, two groups must be formed, the first embracing those that are most benefitted by the drug, and the second, those that receive little aid from it. The first group embraces those who have a temperature of 104° F. (40° C.) and who are well nourished; the second, those that have a moderate temperature, 102° F. (39° C.) or less, and who are not well nourished. Both classes are aided by antipyrin treatment, but the former much more so. Usually after the administration of a dose, the little patients break out in a profuse perspiration, the temperature falls, they sleep for an hour or so, and awake with a feeling of well-being. The cough is usually looser, and the breathing easier. The dosage recommended is for children under five and over two years, twelve or thirteen grains (0.9 gms.). Its effect will continue from twelve to fifteen hours, and, therefore, two doses must be given daily. To a child two years old or less, seven grains (0.5 gms.) may be given, but only one dose will be needed in the twenty-four hours. During convalescence, one-half of the above-mentioned quantities are given at a time. The treatment of acute bronchitis among children by antipyrin, as recommended by Friedlander, does not include the simultaneous use of expectorants.—*The Epitome.*

